# Cholestasis after Kasai operation predicts portal hypertension in native liver survivors of biliary atresia: a multicenter study

**DOI:** 10.1007/s00383-024-05775-0

**Published:** 2024-07-17

**Authors:** Patrick Ho Yu Chung, Toshio Harumatsu, Yoichi Nakagawa, Koichi Tsuboi, Edwin Kin Wai Chan, Michael Wai Yip Leung, Fanny Yeung, Mitsuru Muto, Takafumi Kawano, Hizuru Amano, Chiyoe Shirota, Hiroki Nakamura, Hiroyuki Koga, Go Miyano, Atsuyuki Yamataka, Satoshi Ieiri, Hiroo Uchida, Kenneth Kak Yuen Wong

**Affiliations:** 1 Division of Paediatric Surgery, Department of Surgery, School of Clinical Medicine, Li Ka Shing Faculty of Medicine, The University of Hong Kong, Hong Kong, HKSAR People’s Republic of China; 2 Department of Pediatric Surgery, Kagoshima University, Kagoshima, Japan; 3 Department of Pediatric Surgery, Nagoya University Graduate School of Medicine, Nagoya, Japan; 4 Department of Pediatric General and Urogenital Surgery, Juntendo University School of Medicine, Tokyo, Japan; 5 Division of Pediatric Surgery and Pediatric Urology, Department of Surgery, Prince of Wales Hospital, The Chinese University of Hong Kong, Hong Kong, HKSAR People’s Republic of China; 6 Department of Surgery, Hong Kong Children’s Hospital, Hong Kong, HKSAR People’s Republic of China

**Keywords:** Biliary atresia, Kasai portoenterostomy, Portal hypertension, Cholestasis, Hypersplenism

## Abstract

**Purpose:**

This study evaluated portal hypertension (PHT) and its predictors among native liver survivors (NLS) of biliary atresia (BA) after Kasai portoenterostomy (KPE).

**Methods:**

This was a multicenter study using prospectively collected data. The subjects were patients who remained transplant-free for 5 years after KPE. Their status of PHT was evaluated and variables that predicted PHT were determined by regression analysis and receiver operating characteristic (ROC) curve.

**Results:**

Six centers from East Asia participated in this study and 320 subjects with KPE between 1980 to 2018 were analyzed. The mean follow-up period was 10.6 ± 6.2 years. At the 5th year after KPE, PHT was found in 37.8% of the subjects (*n* = 121). Patients with KPE done before day 41 of life had the lowest percentage of PHT compared to operation at older age. At 12 months after KPE, PHT + ve subjects had a higher bilirubin level (27.1 ± 11.7 vs 12.3 ± 7.9 µmol/L, *p* = 0.000) and persistent jaundice conferred a higher risk for PHT (OR = 12.9 [9.2–15.4], *p* = 0.000). ROC analysis demonstrated that a bilirubin level above 38 µmol/L at 12 months after KPE predicted PHT development (sensitivity: 78%, specificity: 60%, AUROC: 0.75).

**Conclusions:**

In BA, early KPE protects against the development of PHT among NLSs. Patients with persistent cholestasis at one year after KPE are at a higher risk of this complication. They should receive a more vigilant follow-up.

**Level of evidence:**

Level III

## Introduction

Even though liver transplant (LT) is not always necessary for patients with biliary atresia (BA) after Kasai portoenterostomy (KPE) due to normal or near-normal liver function, disease-related complications can impair the well-being of the native liver survivors (NLS). Among these, portal hypertension (PHT) is the most common and potentially life-threatening problem. It has been estimated that more than half of BA survivors will suffer from this distressing complications which also increases the likelihood of LT in future [1, 2]. Most previous studies focused on the improvement of transplant-freed survival but there is a paucity of reports that evaluate PHT among NLS. Early prediction of this complication is challenging [3, 4]. As a result, most centers adopt a ‘onesize-fits-all’ protocol to follow-up BA patients without attending to  the individual’s risk. The purpose of this study was to evaluate PHT among NLS at medium-term follow-up after KPE. Moreover, we analyzed factors that may help to predict the development of PHT.

## Methods

### Subjects

This was a cross-sectional, multicenter study and subjects were recruited from six pediatric surgical centers in Hong Kong, Kagoshima, Nagoya and Tokyo. All participating centers have treated BA for more than 30 years. The data of BA patients who underwent KPE between 1980 to 2018 were reviewed. The study time point was from the 5th year after KPE and patients who were recorded dead or had received LT within 5 years after KPE were excluded. In addition, those with incomplete follow-up data were also excluded. In all centers, BA was diagnosed by surgical exploration to reveal an atretic bile duct. Operative cholangiogram and liver biopsy were performed as adjuvant tests to confirm the diagnosis. KPE was performed by either conventional or laparoscopic approach at the discretion of the operating surgeon. Post-operatively, adjuvant steroids were given according to the protocols of individual centers.

### Study design

Demographic information and peri-operative data were retrieved from medical charts. Clinical outcome including survival status, presence of PHT, liver biochemistry and platelet count based on follow-up data at the study time point were collected. In this study, PHT was the primary outcome. It was defined as the presence of splenomegaly confirmed by radiological studies and thrombocytopenia (platelet count less than 100 X 10^9 L). The age at operation, operative approach, adjuvant steroid usage and jaundice clearance (JC) (defined as serum bilirubin  < 20 µmol/L) after KPE were analyzed for their association with PHT. This study has been approved by the institutional board of the respective units and was performed in accordance with the ethical standards in the Declaration of Helsinki (IRB approval numbers: Hong Kong: UW20-156 / Nagoya: 2021-039123264 / Kagoshima: 27-119 / Juntendo: 20-307).

### Statistical analysis

Scientific analysis was performed with a standard statistical package (Windows, version 26.0; SPSS Inc., Armonk, NY, USA). Data between subjects with PHT  + ve and −ve were compared. Categoric variables were compared with Chi-square test. Continuous variables were presented as median (interquartile range) and compared with Mann–Whitney test. The association of clinical variables with PHT was tested by regression analysis. Independent variables that were considered significant were examined by area under receiver operating characteristic curve (AUROC) analysis to determine the accuracy of prediction. In this cohort, an AUROC  ≥ 0.70 was considered as a clinical model with reasonable accuracy. The cut-off values of predictive variables were determined by the highest sum of sensitivity plus specificity and the positive predictive value (PPV) as well as negative predictive (NPV) values were also calculated. In all analyses, a *p*-value of  < 0.05 was considered to be statistically significant.

## Results

### Study population

Between 1980 to 2018, there were a total of 574 BA patients that had undergone KPE. After excluding patients who died or received LT within 5 years after KPE (*n* = 228) and incomplete data (*n* = 26), 320 subjects were included. In this cohort, male was slightly predominant (male:female = 1.35:1). Two hundred seventy-seven patients underwent conventional open KPE while 43 patients underwent laparoscopic KPE. The mean age at KPE was 54.8 ± 10.9 days and the majority (25.9%, *n* = 83) had KPE between day 51 to day 60 of life. The mean follow-up period was 10.6 ± 6.2 years. Post-operatively, 80.6% of patients (*n* = 258) were given adjuvant steroid according to the protocol of individual units. At 12 months after KPE, 63.4% (*n* = 203) of patients achieved JC (Table 1).

**Table 1:** Demographic data and clinical outcomes of 320 native liver survivors with KPE performed between 1980 to 2018

Patient characteristics	Number (%) or mean ± SD
Sex	
Male	184 (57.5%)
Female	136 (42.5%)
Follow-up period	10.6 ± 6.2 years
Age of KPE (days)	
< 41	40 (12.5%)
41 to 50	53 (16.6%)
51 to 60	83 (25.9%)
61 to 70	66 (20.6%)
> 70	78 (24.3%)
Mean	54.8 ± 10.9
Surgical approach	
Open	277 (86.6%)
Laparoscopic	43 (13.4%)
Use of adjuvant post-operative steroid	
Yes	258 (80.6%)
No	62 (19.3%)
Jaundice clearance	
12 months	203 (63.4%)
Latest follow-up	241 (75.3%)
Portal hypertension (PHT)*	121 (37.8%)
Thrombocytopenia*	130 (40.6%)
Platelet count (× 10^9/L)*	155.3 ± 27.5
Albumin (g/L)*	40.8 ± 5.6
Presence of OGV^#^	81 (62.3%)

^*^ Based on data collected at the study time point. ^#^ Analysis based on 130 patients who have undergone upper endoscopy at a mean age of 12.6 ± 4.8 years

### Prevalence of PHT and related complications at 5 years after KPE

At 5 years after KPE, PHT was found in 37.8% of subjects (*n* = 121) and the mean platelet count was 155.3 ± 27.5 × 10^9 /L. One hundred and thirty patients have received at least one upper endoscopy and 81 patients (61.3%) were found to have oesophago-gastric varices (OGV) (Table 1). PHT + ve subjects had a lower platelet count (PHT + ve vs −ve = 71.2 ± 12.8 × 10^9/L vs 230.1 ± 82.9 × 10^9/L, *p* = 0.011). As expected, there were more patients suffered from thrombocytopenia among PHT + ve subjects (Table 2).

**Table 2:** Comparisons between PHT positive (+ ve) and PHT negative (- ve) subjects, including the odds ratios of the association, when appropriate, between different clinical variables and PHT

	PHT +ve (n = 121)	PHT −ve (n = 199)	*p* value	*OR (95% CI)*	*p* value
Mean age at KPE (day)	62.7 ± 17.1	59.2 ± 20.8	*0.105*		
Mean age of subjects (year)	***14.1 ± 8.9***	***9.7 ± 9.7***	***0.019***		
Mean platelet count (10^9/L)	***71.2 ± 12.8***	***230.1 ± 82.9***	***0.011***		
No. of patients with thrombocytopenia	***81***	***35***	***0.000***		
Albumin (g/L)	40.3 ± 5.8	41.3 ± 5.4	0.434		
No. of patients achieving jaundice clearance at 12 m post-KPE	***76***	***160***	***0.000***	***12.9 (9.2–15.4)***	***0.000***
Bilirubin level (µmol/L) at 12 m post-KPE	27.1 ± 24.7	12.3 ± 7.9	0.000		
No. of patients with conventional open surgery (n = 268)	101	176	0.354	0.8 (0.1–1.4)	0.352
No. of patients receiving adjuvant steroid (n = 258)	96	162	0.848	0.4 (0.0–0.9)	0.848

Values in bold and italics are clinical variables which have statistical significance between PHT +ve and PHT -ve patients

### The association of different clinical variables with PHT

#### Peri-operative variables

**Fig. 1:**
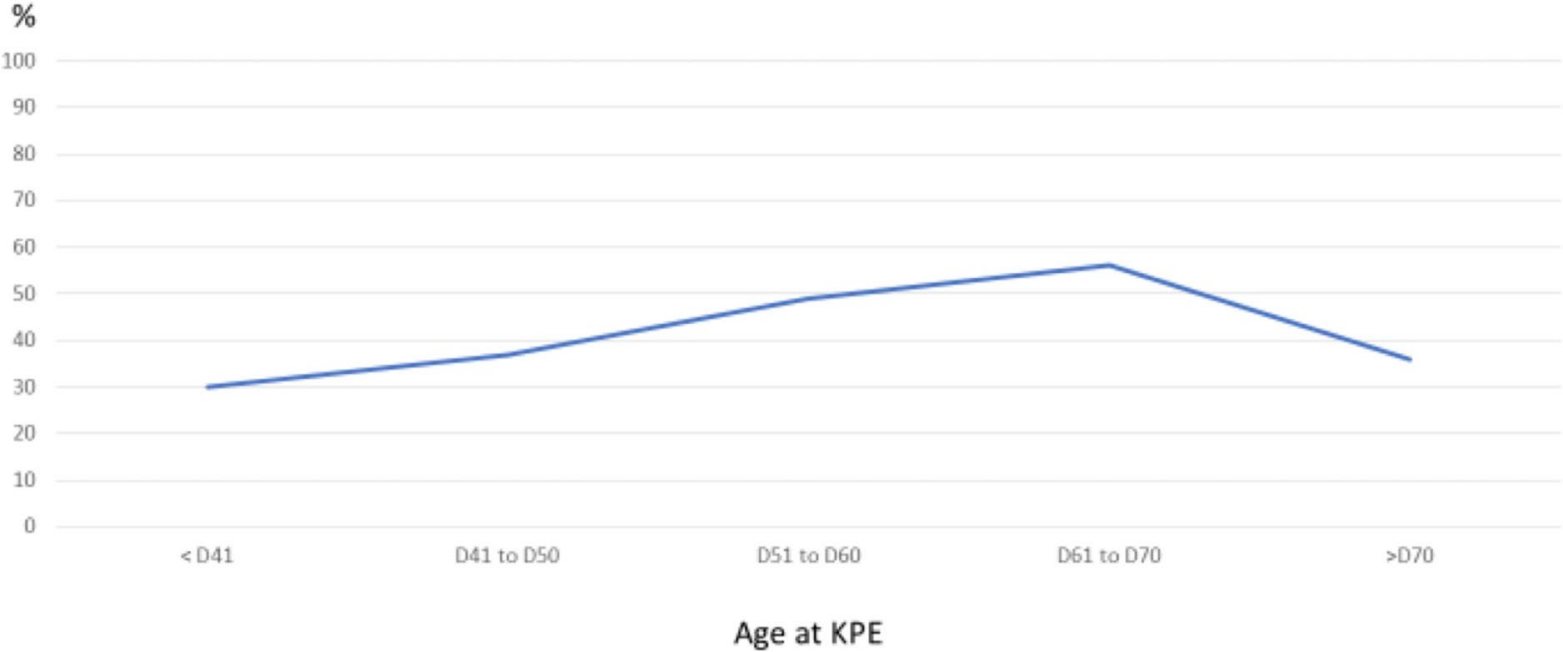
The percentage of PHT at the 5th year after KPE according to the age of operation

Regarding the impact of the age of KPE on PHT, a linear relationship was observed during the initial phase until day 70 of life (Fig. 1). However, there was no significant difference when the mean age of KPE between PHT + ve and -ve was compared (PHT + ve vs −ve = 62.7 ± 17.1 vs 59.2 ± 20.8, *p* = 0.105). The other peri-operative factors including the operative approach and the use of adjuvant steroid were also not significantly associated with PHT (*p* = 0.354 and 0.848, respectively). The results of the analyses are shown in Table 2.

### Post-operative jaundice clearance

**Fig. 2:**
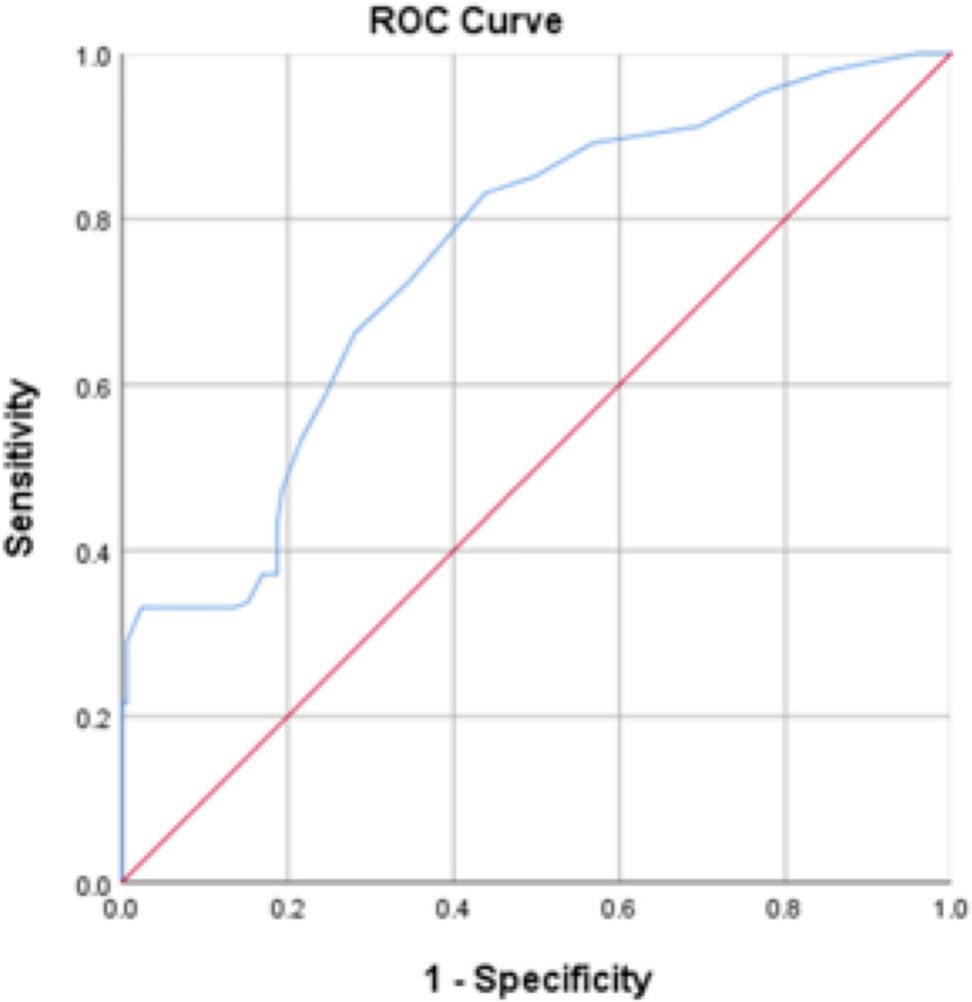
ROC curve analysis of the accuracy of prediction/detection of PHT by bilirubin level at 12 months after KPE

Among NLS, JC at 12 months after KPE was associated with a significantly lower percentage of PHT (62.8% vs 80.8%, *p* = 0.000.) When we evaluated the post-KPE bilirubin level, it was higher in PHT + ve subjects (27.1 ± 24.7 vs 12.3 ± 7.9 µmol/L, *p* = 0.000). Multivariate regression analysis revealed that the persistent cholestasis at 12 after KPE was associated with a higher chance of PHT (OR = 12.9 [95% CI 9.2–15.4], *p* = 0.000) (Table 2). ROC curve analysis (Fig. 2) demonstrated that a bilirubin level over 38 µmol/L at 12 months after KPE predicted the development of PHT (sensitivity: 78%, specificity: 60%, PPV: 63%, NPV: 76%, AUROC: 0.75 [95% CI 0.70 – 0.81]) (Table 3).

**Table 3:** The sensitivity, specificity, positive predictive value and negative predictive value of bilirubin level at 12 months after KPE in predicting of PHT among NLSs

Bilirubin level	PHT + ve	PHT −ve	Total	Sen	Spec	PPV	NPV
> 38 µmol/L	94	80	174	78%	60%	63%	76%
< = 38 µmol/L	27	119	146				
Total	121	199	320				

## Discussion

In BA patients, despite the fact that LT is not immediately required in many after KPE, up to 50% of them will suffer from residual complications owing to progressive liver fibrosis [5]. Since the measurement of portal pressure requires invasive venous cannulation, the presence of splenomegaly is broadly taken as the major diagnostic criterion of PHT [4]. However, a prediction before its onset is difficult due to the lack of reliable indicators.

PHT can develop as early as 1 to 2 years after KPE but in most cases, it takes a few years to become clinically significant. In this study, we were particularly interested to evaluate PHT among post-KPE patients who did not require LT. It was because they are frequently labeled as the ‘cured’ ones since PHT could be asymptomatic. As a result, they often receive a less vigilant follow-up until complications happen. In this cohort, approximately 38% of NLS suffered from PHT at a medium follow-up period and this finding is consistent with previous reports. Splenomegaly may lower the platelet count, but approximately one-third of PHT  + ve subjects did not suffer thrombocytopenia. Moreover, although the platelet count of PHT  + ve subjects was significantly lower, the value is unlikely to cause severe bleeding tendency. We believe that serial monitoring of platelet count is necessary but in the majority of the cases, an aggressive treatment such as splenectomy or LT is not indicated. In symptomatic cases with marked thrombocytopenia, splenic artery embolization has been proposed but further validation by large-scaled study is required [6]. While splenomegaly maybe clinically unremarkable, the correlation of splenomegaly with disease progression has been shown. Attention should therefore be given to the spleen size as an indirect reflection of the disease progress [7]. OGV, the other manifestation of PHT, deserves more attention even when it is asymptomatic. Variceal bleeding can be life-threatening especially when thrombocytopenia co-exists but unfortunately, only 70% of pediatric centers performed surveillance screening for OGV according to an international survey [8]. Due to the inconsistent policy of surveillance endoscopy in the participating units, we were not able to perform an in-depth analysis on the development of OGV. Spleen stiffness has been reported to predict OGV but this measurement required a sophisticated and expensive equipment that is not widely available [9, 10]. Non-invasive biomarkers such as platelet count and spleen size have been studied for their predictive values with a reasonably good accuracy [11].

To identify variables that may predict PHT development, we analyzed variables that have been reported to influence the survival outcomes. The timing of KPE has been extensively studied for its impact on the drainage rate and transplant-free survival. In most studies, an favorable outcome is linked to an early KPE [12]. Figure 1 shows a trajectory that an early KPE was associated with a lower prevalence of PHT. We believe the distortion of this relationship towards the end of the curve could be related to patient selection. As late KPE could lead to a higher chance of liver failure, we postulated that some sicker patients have been transplanted soon after KPE and therefore were excluded. In a recent publication, we reported the adverse survival outcome for KPE after day 70 of life [13]. Adding together, a collaborative effort should be made to ensure KPE can be performed not later than 2 months of age.

The operative approach is another prognostic factor that is frequently studied. Early systematic reviews recommend KPE should be performed in the conventional open manner but conflicting results were obtained in the most recent review that favored laparoscopic approach [14–16]. Our result demonstrated that operative approach was not a significant factor to influence the development of PHT. Nonetheless, the variations in surgical technique across different surgeons might be confounding.

For adjuvant medications in BA, steroids have been widely used for 20 to 30 years in Hong Kong and Japan. Their beneficial effects on the KPE drainage and transplant-free survival rate have been reported [17]. However, their impact on the development of PHT was not obvious in this study. Steroids are usually given as an intensive therapy during the early post-operative period only, and therefore, the effect may not be long-lasting to prevent on-going liver fibrosis after KPE. To understanding the true impact of steroids on PHT, a prospective study will be required. Anti-fibrotic agents have been proposed in adult patients with liver diseases [18]. However, this is not a generalized practice in children due to its inconsistent efficacy.

Failure of JC has been shown to adversely affect the long term survival. Here we also revealed that it was a risk factor for future development of PHT. We postulate that the resolution of jaundice after KPE alleviates the intrahepatic cholangiopathy and the subsequent liver fibrosis. This finding is also compatible to the result obtained in a study that reported the association between hyperbilirubinemia and PHT in adults [19]. On account of this, we deliberately attempted to determine the level of bilirubin that may predict PHT. The risk of PHT is diminished only when the post-KPE bilirubin could be below 38 µmol/L. While an effective strategy to lower the bilirubin is pending, patients with persistent cholestasis for more than a year after KPE should be closely monitored for PHT complications. Even though they may not require liver transplant due to relatively normal liver biochemistry, an active search for PHT complications is still recommended. In this regard, the optimal level of post-KPE bilirubin level to define a ‘successful’ operation in BA will need further prospective study to determine.

Compared to previous studies with similar objective, the major strength of our study is the large sample size and the data were collected from multicenters that are experienced in managing BA. The finding is readily applied to patients in different regions in Asia. Nevertheless, we acknowledged that some unmeasurable factors such as the variation of operative technique as well as peri-operative management inevitably affected the analysis. Second, this was a cross-sectional analysis and subjects were examined at a single-time point without longitudinal data. Third, our study evaluated NLS only and transplanted patients who might have been suffering from concomitant PHT were excluded. Lastly, portal venous measurement was not performed and PHT was diagnosed based on clinical manifestations only. The true prevalence of PHT might have been inaccurately estimated. By defining PHT as the presence of splenomegaly, we might have over-estimated this condition. On the other hand, this looser definition put us on the safe side to include all patients even with minor PHT symptoms. The findings of this analysis would therefore help to formulate a protocol that offers a more aggressive protection.

Despite the aforementioned limitations, we believe the findings are informative. Our results demonstrated that the majority of BA patients who, despite remaining transplant-free, are not completely ‘cured’. Effort should be made to facilitate an early KPE for its protection against PHT, in addition to the survival benefit as previously reported. Persistent cholestasis after KPE is a significant predictor for PHT in NLSs of BA especially among those with bilirubin level higher than 38 µmol beyond the first year of KPE. They should be informed about the possibility of PHT in future even if they can remain transplant-free.

## Abbreviations

BA: Biliary atresia
KPE: Kasai portoenterostomy
JC: Jaundice clearance
LT: Liver transplant
NLS: Native liver survival
OGV: Oesophago-gastric varices
PHT: Portal hypertension
ROC: Receiver operating characteristic
